# When size matters: A novel compact Cas12a variant for in vivo genome editing

**DOI:** 10.1371/journal.pbio.3002637

**Published:** 2024-07-17

**Authors:** Felix Bubeck, Dirk Grimm

**Affiliations:** 1 Department of Infectious Diseases/Virology, Section Viral Vector Technologies, Medical Faculty, Heidelberg University, Heidelberg, Germany; 2 BioQuant, BQ0030, Heidelberg University, Heidelberg, Germany; 3 Faculty of Engineering Sciences, Heidelberg University, Heidelberg, Germany; 4 German Center for Infection Research (DZIF) and German Center for Cardiovascular Research (DZHK), partner site Heidelberg, Heidelberg, Germany

## Abstract

Adopting Cas12a nucleases for AAV-based gene therapy has been difficult due to their large size. A new study in PLOS Biology characterizes and engineers a new Cas12a nuclease variant that is compatible for in vivo genome editing via an all-in-one AAV delivery system.

## Teaser

A new study characterizes and improves a novel small Cas12a variant before adapting it for in vivo genome editing by delivery via adeno-associated virus (AAV) vectors, showcasing the potential of small CRISPR systems and their compatibility with viral vectors.

**Fig 1:**
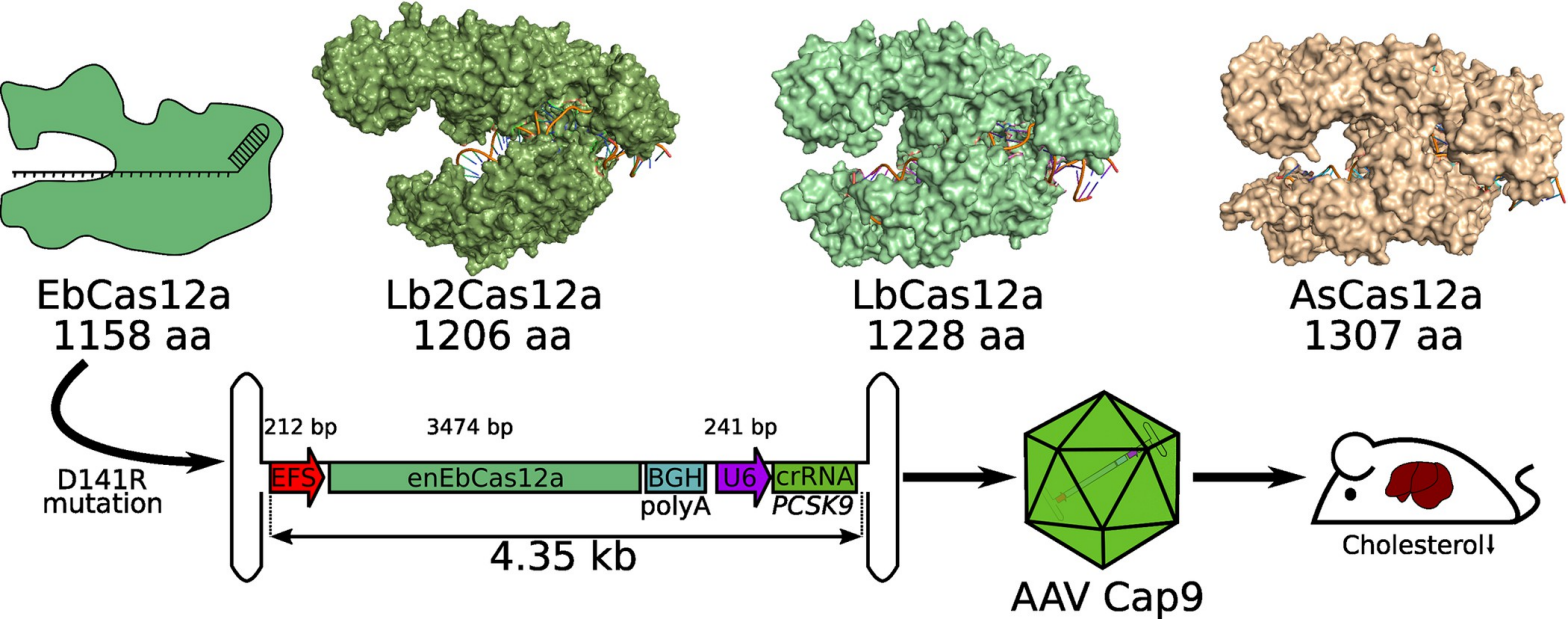
EbCas12a-mediated gene editing with all-in-one AAV vectors. The authors of the new study characterized the novel EbCas12a, which is smaller than other commonly used Cas12a variants such as Lb2Cas12 (PDB: 8I54), LbCas12a (PDB: 5XUT), and AsCas12a (PDB: 5B43). The D141R point mutation could further enhance the activity of EbCas12a, generating the variant enEbCas12a, which was then incorporated into an AAV vector genome. In this context, enEbCas12a expression is driven by an EFS promotor. In addition, the vector genome also contains a crRNA expression cassette driven by a U6 promotor. Altogether, the components of the EbCas12a system are well below the 4.8-kb packaging limit of AAVs, enabling successful packaging in the AAV9 capsid. By targeting the *PCSK9* gene in hepatocytes with the “all-in-one” enEbCas12a vector, a decrease in serum cholesterol could be observed in mice.

For gene therapy via adeno-associated viral (AAV) vectors to be successful, the vector genome size is a major constraint as it prevents the packaging of large transgenes into the icosahedral viral capsid. In the context of CRISPR-based genome editing, a variety of compact Cas variants have been described such as Cas12j (CasPhi) or Cas12f (CasMINI) [1,2], which could be employed for in vivo genome editing by delivery via AAVs. In the study by Wang and colleagues [3], a novel compact Cas12a variant from *Erysipelotrichia bacterium* (EbCas12a) is characterized that, when improved by a point mutation (enEbCas12a), displays similar editing efficiencies as other Cas12a variants [3]. Notably, the coding sequence for EbCas12a is around 150 bp smaller than the next larger characterized Cas12a variant (Fig 1), facilitating its accommodation in an “all-in-one” AAV vector providing Cas12a and crRNA on a single AAV template. By using a single vector administration, the authors then demonstrate the ability of the novel AAV-enEbCas12a vector to mediate in vivo genome editing, thereby adding a new entry to the ever-expanding CRISPR toolbox.

Initially, the authors characterized EbCas12a via in vitro cleavage assays and determined TTTV (V = G, C, or A) as PAM sequence, which is analogous to other reported Cas12a systems. Next, EbCas12 was shown to be functional in cultured mammalian cells, as indicated by the cleavage of two reporters and various genomic loci. However, in comparison with other Cas12a variants, such as the commonly used AsCas12a and LbCas12a, EbCas12a lacked in efficiency. Therefore, as done previously to relax PAM sequence constraints and enhance the editing efficiency of AsCas12a [4], the authors substituted a single amino acid in EbCas12a that establishes PAM-proximal DNA contacts, resulting in the variant enEbCas12a. Indeed, this single point mutation improved editing efficiency of genomic loci around 2-fold. Yet, at the same time, the relaxation of the PAM sequence constraints may increase the risk of off-target editing by enEbCas12a. To examine this concern experimentally, a genome-wide profiling of off-target editing events was performed. Notably, the number of detected off-target sites was similar to the commonly used As/LbCas12a variants, while the on-target efficiency was comparable as well. Together, this characterizes the newly engineered enEbCas12a as similarly efficient and specific as the other Cas12a variants.

Next, to enable in vivo genome editing, the enEbCas12a coding sequence was incorporated into an AAV vector genome and packaged in an AAV9 capsid. AAVs are constrained by their small packaging capacity, limited to around 4.8 kb for the entire gene expression cassette. Importantly, though, the relatively small size of enEbCas12 allowed packaging of an EFS promotor-driven cDNA together with U6 promotor-controlled crRNA expression cassette in a single AAV vector genome. It is noteworthy that the combined size of the Cas12a and crRNA expression system is only around 4.4 kb, still leaving space for additional regulatory sequences or the use of larger promotor sequences. As AAV vector-mediated delivery of enEbCas12a resulted in efficient editing in cultured mammalian cells, vectors targeting the *PCSK9* gene were subsequently administered in mice by intravenous injection. PCSK9 is secreted by hepatocytes and is involved in LDL-receptor degradation, thereby affecting LDL-cholesterol levels. After enEbCas12a-mediated disruption of *PCSK9*, a mild but significant reduction in cholesterol could be observed, along with moderate genome editing levels with around approximately 9% indel frequency. Curiously, the indel frequency 21 days after the AAV injection was lower than after 14 days, which may either indicate a regeneration of nonedited cells or the removal of transduced/edited cells. This could hint at an immune response against the AAV vector (genome or capsid) or against the Cas12a of bacterial origin, both of which are known challenges that the scientific community is currently attempting to overcome [5].

Delivery of transgenes via AAV9 particles is commonly employed due to the wide tropism of this viral serotype, which targets a variety of tissues in the body after systemic administration. Still, since the *PCSK9* gene is expressed in hepatocytes, thus use of a capsid with high transduction of these cells and low transduction levels in other off-target cells may be beneficial. Ideally, this strategy would improve the effect of serum cholesterol reduction while simultaneously reducing potential deleterious effects from delivery of functional Cas12a into other tissues. Even though targeting of murine hepatocyte by AAV9 is well described, other viral serotypes, such as AAV7 or AAV8, may further improve the efficiency, specificity, and, thus, safety of in vivo gene editing by increasing hepatocyte transduction and reducing systemic delivery [6]. Furthermore, engineered capsids with an even narrower tropism but efficient transduction and reduced immunogenicity could additionally improve the outcome of such genetic interventions [7].

Collectively, this work represents a pivotal first step by introducing the small EbCas12a and its derivative enEbCas12a to the CRISPR toolbox, but substantial work is still required to properly characterize this ortholog, including an assessment of its *trans*-cleavage activity. Moreover, enEbCas12a will have to compete with other, in some cases even smaller Cas variants displaying their own sets of characteristics that may favor specific applications. Of note, while the commonly used Cas9 system generates blunt end cuts or single-nucleotide overhangs, Cas12a results in a staggered cleavage [8], which may promote directional insertion of exogenous DNA fragments into a target sequence. Another major advantage of the Cas12a system is the ability to autonomously process its crRNA from a crRNA array without relying on accessory proteins [9]. This will prove useful when targeting multiple sites at the same time, especially in scenarios that merely rely on target disruption such as antiviral CRISPR therapy of DNA viruses or integrated retroviral genomes. In these cases, the possibility to concurrently disrupt multiple sites with a single vector may reduce the risk of viral evasion and thus render an “all-in-one” Cas12a system a particularly attractive therapeutic option. Future work may also include the generation of nickase enEbCas12a variants that may be used as a basis for Base or Prime Editors, where the compact size of this Cas protein could facilitate vector-mediated delivery.

Lastly, the advantages of this novel small Cas12a variant may not only benefit genome editing but could also be explored for in vivo CRISPR-based transcriptional modulation. To this end, it should be appealing to inactivate the DNA cleavage activity of Cas12a by mutation, generating an RNA-guided DNA-binding system. When fused to a transcriptional activator or repressor, a targeted induction or repression, respectively, of transcription at a specific genomic locus could be achieved [10]. The small size of the enEbCas12a is again beneficial in this context as it facilitates delivery via a single AAV vector and thus increases the applicability of this tool. In fact, it could not only be used for therapeutic reactivation of silenced genes, but the combination of transcriptional modulation and the ability to target multiple genomic loci with a crRNA array may also be harnessed for perturbation of gene expression networks or for cellular reprogramming with a single AAV vector.
